# The economic burden of cancers attributable to smoking in Korea, 2014

**DOI:** 10.18332/tid/102673

**Published:** 2019-02-28

**Authors:** Thi Xuan Trinh Nguyen, Minji Han, Jin-Kyoung Oh

**Affiliations:** 1 Department of Cancer Control and Population Health, National Cancer Center Graduate School of Cancer Science and Policy, Goyang, Republic of Korea; 2 National Cancer Control Institute, National Cancer Center, Goyang, Republic of Korea

**Keywords:** cancer, smoking, economic burden, Korea

## Abstract

**INTRODUCTION:**

Associations between smoking, cancer and mortality are well established. Although cancer mortality rates have decreased in recent years, the economic burden of smoking-related cancers continues to increase. This study investigates the economic costs of cancers related to smoking in Korea in 2014.

**METHODS:**

Cancer patients were identified through National Health Insurance Services medical claims with ICD-10 cancer codes. We multiplied the costs by the population attributable fraction for each type of cancer and calculated direct and indirect costs, where direct costs comprise direct medical and non-medical costs of inpatients and outpatients, and indirect costs include estimates of future income loss due to premature death, productivity loss during hospitalization and outpatient visits, and job loss.

**RESULTS:**

In 2014, there were 79297 smoking-related cancer patients, accounting for 8.47% of all Korean cancer cases for that year. The direct cost of cancers due to smoking was approximately 595 million USD, whereas indirect costs were much higher, at nearly 2.2 billion USD. The average expenditure of a typical patient was 34815 USD. Lung, liver and stomach cancers were most prevalent and represented the most significant share of the economic burden, whereas the largest per-patient spending was for pancreatic, liver, and lung cancers. Lung, liver and stomach cancers had the highest economic impact on men, while lung, liver and ovarian cancers had the most significant economic impact on women.

**CONCLUSIONS:**

It is imperative that more stringent steps be taken to reduce the huge economic burden of cancers linked to smoking.

**ABBREVIATIONS:**

IARC: International Agency for Research on Cancer, PAF: population attributable fraction, NHIS: National Health Insurance Services, ICD-10: International Statistical Classification of Diseases and Related Health Problem 10th Revision, GDP: Gross Domestic Product, DALYs: Disability-Adjusted-Life-Years, WHO: World Health Organization, FCTC: Framework Convention on Tobacco Control.

## INTRODUCTION

Smoking is associated with a variety of diseases and poses a major public health threat. Tobacco smoking, the most common form of smoking, encompasses over 4000 chemicals, of which more than 70 are known to be carcinogenic^1^. Causal relationships between cigarette smoking, increased all-cause mortality, and cancer-specific mortality are well established^2^. Based on present consumption patterns, approximately 450 million adult smoking-related deaths will occur between 2000 and 2050^3^; half of the cases are predicted to die between the ages of 30 and 69 years. Although the number of smokers experienced a strikingly large increase over recent years as a result of global population growth^4^, since 1980 smoking prevalence has declined sharply in men and women worldwide. In particular, from 1990 to 2015 global smoking exposure decreased by more than 25%^5^. Despite the decreased exposure, smoking still plays a major part in the Disability-Adjusted-Life-Years (DALYs) outcome^5^. Intensive efforts have been produced in the world’s fight against tobacco since MPOWER was first deployed to help implement the World Health Organization’s Framework Convention on Tobacco Control (WHO FCTC), with around two-thirds of countries (121 of 194) - consisting of 63% of the global population - starting to put at least one MPOWER measure into practice at the greatest level of achievement^6^. Although cancers and overall mortality from smoking have decreased significantly in highly developed countries, they are set to rise worldwide unless current smokers, most of whom live in low- and middle-income countries, stop smoking before or during middle age. The detrimental effect of smoking on society and healthcare system economies is substantial^7^; medical expenses arising from smoking-attributed diseases reached 5.7% of global healthcare spending in 2012, which is equal to 1.8% of the world’s gross domestic product (GDP). In some developing countries, smoking-related healthcare spending accounted for 40% of all healthcare spending, which underscores the overwhelming burden these countries carry^8^.

In Korea, the smoking rate among men is relatively high compared to other developed countries, even though it declined from 66.3% in 1998 to 40.7% in 2016^9^. The smoking rate among women was 6.4% in 2016 and has not changed significantly since 1998. To reduce smoking prevalence, Korea started to promote smoking ban policies in 1995 and efforts to reinforce and expand these programs have continued^10^. After Korea ratified the FCTC in 2005, the required policies incrementally strengthened tobacco control. According to the monitoring MPOWER in 2017 and the national progress report on the implementation of the FCTC in 2018, Korea has strengthened tobacco control policies, including tobacco tax increases, mass media campaigns, pictorial warnings on tobacco packs, smoke-free bars and restaurants, and reimbursement for smoking cessation services provided by healthcare facilities. However, the price of cigarettes remains low considering the nation’s income level, and tobacco advertisements, promotion and sponsorship are only partially restricted. Workplace smoke-free policies are limited to large companies. Only monitoring of tobacco control policies and smoking cessation services are at a high level in Korea^6,11,12^.

Tobacco smoke was ranked as the third leading cause of DALYs in Korea in 2013^13^. Cancer, one of the diseases most initiated by smoking, is a leading cause of death in Korea and has a high incidence rate (259 cases per 100000 persons in 2014)^14^. The negative health impact of smoking can be measured by quantifying smoking-associated illnesses, premature mortality, and productivity losses in monetary terms^15^. In Korea, several studies have been conducted to examine the burden of active and passive smoking-attributable diseases; however, most of them employed the DALY measure to express disease burden^10,16-18^. Two studies have examined the economic burden of smoking-associated diseases^19,20^, one of which focused specifically on smoking-associated cancer in 2008^20^. However, cancer incidence and mortality have changed in recent years; all-cancer mortality has decreased since 2002 while cancer incidence increased until 2012 then began to decrease^14^. In this study, we examined more recent data to update estimates regarding the economic burden of smoking-related cancers in Korea.

## METHODS

### Smoking-related cancer types

Major cancers attributable to smoking were identified from the International Agency for Research on Cancer (IARC) evaluation on carcinogenicity^1^ and previous studies^20,21^ and defined according to the International Statistical Classification of Diseases and Related Health Problems 10th Revision (ICD-10): cancers of the oral cavity (C00-C09), pharynx (C10-C14), esophagus (C15), stomach (C16), colorectum (C18-C20), liver (C22), pancreas (C25), larynx (C32), lung (C33-C34), cervix uteri (C53), ovary (C56), kidney (C64), and bladder (C67). This study used the gender-specific smoking-attributable fractions (SAF) for each cancer type in the Korean population from a published study^21^. In brief, sexand cancer-specific population-attributable fractions (PAF) were estimated using the prevalence of ever smoking and secondhand smoking in 1989 among Korean adults, respectively, and the relative risks were estimated from the meta-analysis of studies performed in the Korean population for the case of ever smoking and in the Asian population for passive smoking. National cancer incidence data from the Korea Central Cancer Registry and national cancer mortality data from Statistics Korea for the year 2009 were used to estimate the cancer cases and deaths attributable to tobacco smoking^21^.

The total economic cost of cancers attributed to smoking was measured by multiplying the cost of each cancer site by its respective SAF according to the formula:

*Smoking-attributable costs = Total costs of cancer sites × SAF*

### Data source

Health insurance claims data provided by the National Health Insurance Services (NHIS) were used to estimate prevalence and direct medical costs of smoking-attributable cancers. The NHIS is a mandatory single payer insurance that provides benefits for medical services. All South Korean citizens must either be enrolled in the NHIS (97% of the population) or must be a recipient of medical aid (3%)^22^. The NHIS database contains information on medical aid subjects as well; therefore, it contains information on the Korean population. Currently, the NHIS maintains and stores national records for healthcare use and prescriptions. The NHIS claims data contain details on the cost of care, medical institution attended, income distribution, and residential information for all insurance subscribers^23^.

To estimate direct non-medical costs and indirect costs, data from diverse sources were used including the Korea Health Panel Survey (KHPS)^24^, cause of death statistics and life tables provided by Statistics Korea^25^, and employment rates and wage data provided by the Ministry of Employment and Labor^26^ (See Supplementary Table).

This study used anonymous secondary data and was exempted from review by the Institutional Review Board of the National Cancer Center, Korea (NCC2017-0131). Consent was waived.

### Estimation of economic burden

The economic burden of cancers associated with smoking was evaluated using a prevalence-based approach that targeted existing and newly diagnosed cancer patients. Patients who visited or were hospitalized in medical institutions with smoking-related cancer as a primary diagnosis in 2014 were included in the analysis. In the claims data, the special code V193, which indicates a confirmed cancer diagnosis for expanded benefit coverage, was used to define cancer cases. Given the time-lag between initiating a smoking habit and cancer development, the study population included only those aged 20 years and older^10,17^.

The total economic burden of smoking-attributed cancers was calculated as the sum of direct costs and indirect costs.

*Total costs = Direct costs + Indirect costs*

### Direct costs

Direct costs are costs incurred during the period of medical treatment and include both medical and non-medical costs. The former includes expenditures for inpatient and outpatient medical treatment, and the latter includes transportation costs for travel to inpatient or outpatient centers, and caregiver costs. Indirect costs comprise productivity loss due to premature death, productivity loss incurred during absences following hospitalization and outpatient visits, and job loss arising from unemployment after a cancer diagnosis.

Direct medical care costs were obtained from the NIHS 2014 medical claims records of existing and newly diagnosed smoking-attributable cancers. Non-covered healthcare costs were set at 19.9%, the rate reported by a previous study^27^. Direct non-medical care costs were calculated from transportation costs for all inpatient/outpatient visits plus total payments to caregivers. The one-way transportation fee per inpatient/outpatient visit was determined to be 13.7 and 3.7 USD, respectively, by analyzing raw data from the 2014 KHPS^28^. These figures were multiplied by the number of visits to obtain transportation costs. Based on the 2014 KHPS, the estimated daily wage of caregivers was 57.7 USD and had a use rate of 67% among inpatients only^28^. Caregivers’ costs were divided into costs incurred during inpatient and outpatient care. Inpatient care costs were calculated under the assumption that each inpatient was accompanied by a healthcare guardian for the entire day; thus, the cost was determined by combining the daily caregiver payment with the number of days hospitalized and the nursing use rate. In addition, costs paid by patients aged 65 years or older were calculated by multiplying the number of outpatient visit days by one-third of the daily caregivers’ wage (See Supplementary Table).

Formulae given below have been adapted from Lee et al.^29^. The direct costs, DC, were calculated from:

DC =∑_i_ ∑_j_ ∑_y_ {(1 + α) *IP_ijy_* + (1 + α) *OP_ijy_*} × *PAF_iy_*+ ∑_i_ ∑_j_ ∑_y_ {(*IV_ijy_* × 2*TIV*) + (*OV_ijy_* × 2*TOV*)} × *PAF_iy_* + ∑_i_ ∑_j_ ∑_y_ {(*IV_ijy_* × *CGR* × *C*) + (*OV’_ijy_* × *CGR*/3)} × *PAF_iy_*.

where, the summations are over gender (i), age (j) and cancer type (y), IP is total treatment amount for inpatients in NHIS data, OP is total treatment amount for outpatients in NHIS data, α is the proportion between non-coverage and coverage rate (in this study, α = 19.9/80.1), IV is the number of days of inpatient visits, OV is number of days of outpatient visits, TIV is cost per one-way trip to hospitals among inpatients, TOV is cost per one-way trip to hospitals among outpatients, CGR is caregivers’ average wage per day, C is use rate, OV’ is number of days of outpatient visits among those aged 65 years and above and PAF is population attributable fraction.

### Indirect costs

Indirect costs were broken down into future income loss, productivity loss and job loss:

*Indirect costs = Future income loss + Productivity loss + Job loss*

To identify costs associated with premature death, a human capital approach was applied, which considered the potential earnings for each deceased person from the year of their death until the end year of their average life expectancy. Productivity loss following premature deaths was calculated using data from Cause of Death Statistics^25^; employment rates and annual average salaries by sex and age were provided by the Ministry of Employment and Labor^26^. Future expected incomes were converted into current values by applying a discount rate of 3% per year. To calculate productivity loss resulting from medical institution visits and hospitalization, the total number of outpatient visits and inpatient admission days were collected from the NHIS claims data. These values were combined with age- and sex-specific employment rates and monthly wage data. Job loss was described as productivity loss due to unemployment resulting from a cancer diagnosis. This was estimated by identifying the number of pre-existing and newly diagnosed smoking-attributed cancer cases along with the overall job loss rate for cancer patients in Korea, which was 47%^30^. These figures were then aggregated with employment rates and daily wage data by age and gender to determine the costs arising from unemployment following a cancer diagnosis (Supplementary Table).

The future income loss, FIL, was calculated from:

FIL = ∑_i_ ∑_j_ ∑_t_ $\Sigma_{\text{k}=1}^{\text{n}}$ {*D_ijt_* × *DPAF_iy_* × (*YWij(t+k)* × *Eij(t+k)*)/(1 + *r*)*^k^*}

where, the summation now includes over age at death (t), years (k) with maximum value n the difference between age at death and life expectancy of cohort, D is number of deaths, DPAF is death population attributable fractions, YW is yearly wage, E is employment rate, and r is discount rate. The productivity loss, PL, was calculated from:

PL = ∑_i_ ∑_j_ ∑_y_ {(*IV_ijy_* + *OV_ijy_*/2) × *PAF_iy_* × *E_ij_* × *DW_ij_*}

where DW is daily wage. The job loss, JL, was calculated from:

JL = ∑_i_ ∑_j_ ∑_y_ (*N_ijy_* × *YW_ij_* × *E_ij_* × *L* × *PAF_iy_*)

where N is number of prevalent cancer cases, YW is yearly wage, and L is job loss rate.

A one-way sensitivity analysis was performed by varying the annual discount rate from undiscounted (0%), to discounted (3%), and more discounted (5%), for productivity loss due to premature death. The analyses were conducted using SAS software (SAS Institute Inc., Cary, NC, USA) and Excel 2013. The costs were converted from KRW to USD using the 2014 exchange rate (1 USD = 1091.85 KRW)^31^.

## RESULTS

In 2014, there were 79297 smoking-attributable cancer cases, accounting for 8.47% of the total cancer patients in Korea. Among these patients, the majority of cancer cases due to smoking (nearly 61%) were in the 60 to 79 years age groups (Table 1). Furthermore, around one-third of total cancer cases attributed to smoking had stomach cancer, followed by lung and liver cancers. This finding did not differ by gender.

**Table 1:** The number of smoking-related cancer patients, according to age in years, in Korea, 2014

*Cancer type*	*Gender*	*20−29*	*30−39*	*40−49*	*50−59*	*60−69*	*70−79*	*80*	*Total*
Oral cavity	Men	44	101	287	698	664	447	111	2351
	Women	22	42	76	129	104	107	50	529
Pharynx	Men	16	47	134	369	405	304	74	1349
	Women	<10	<10	24	37	21	19	<10	118
Esophagus	Men	<10	<10	70	556	1080	1090	242	3045
	Women	<10	<10	<10	<10	<10	<10	<10	14
Stomach	Men	24	354	2031	5828	7271	6237	1604	23348
	Women	<10	31	109	181	186	217	91	817
Colorectum	Men	<10	17	71	230	310	274	80	983
	Women	<10	<10	<10	<10	<10	<10	<10	<10
Liver	Men	<10	127	907	3082	3043	2096	521	9786
	Women	<10	10	40	145	215	234	92	738
Pancreas	Men	<10	19	104	326	482	507	159	1599
	Women	<10	<10	<10	<10	10	14	<10	40
Larynx	Men	<10	14	101	691	1232	1158	301	3498
	Women	<10	<10	<10	13	15	28	10	71
Lung	Men	25	131	710	3073	6371	7919	2491	20721
	Women	<10	14	64	213	283	317	143	1036
Cervix uteri	Men	0	0	0	0	0	0	0	0
	Women	<10	12	24	25	16	11	<10	93
Ovary	Men	0	0	0	0	0	0	0	0
	Women	20	34	84	134	80	44	12	408
Kidney	Men	<10	51	147	261	234	163	35	898
	Women	<10	<10	<10	<10	<10	<10	<10	<10
Bladder	Men	16	90	350	1155	2125	2758	1253	7748
	Women	<10	<10	<10	14	23	38	25	107
Subtotal		200	1111	5344	17171	24173	23985	7312	79297

The economic burden from smoking-attributable cancers in Korea in 2014 was approximately 2.76 billion USD, of which around 35.8% of the total costs were contributed by lung cancers, with the second and third largest costs associated with liver and stomach cancers. The same pattern of the top three cancer types held for men, whereas for women, lung, liver and ovarian cancers were associated with the highest expenditures for smoking-attributed cancers. The total cost for an average smoking-related Korean cancer patient in 2014 was 34815 USD. Smoking-related pancreatic cancer cases incurred the highest costs, with 72720 USD spent per case, followed by liver and lung cancers at 53719 and 45459 USD, respectively (Table 2).

**Table 2:** Total costs of smoking-related cancers by cancer type and sex, in Korea, 2014 (Thousand USD)

*Cancer site*	*Gender*	*Direct costs*				*Indirect costs*				*Total*	*Per patient*
		*Medical*	*Transportation*	*Caregiver*	*Subtotal*	*Future income loss*	*Productivity loss*	*Job loss*	*Subtotal*		
Oral cavity	Men	17975	216	1585	19776	48443	5330	26916	80689	100464	42.734
	Women	3103	39	310	3452	726	333	2170	3229	6681	12.619
Pharynx	Men	14245	172	1421	15837	27622	4195	14073	45890	61727	45.764
	Women	995	13	102	1110	372	130	549	1051	2160	18.346
Esophagus	Men	29212	333	3305	32850	69939	6595	21014	97549	130398	42.825
	Women	111	<10	16	131	<10	<10	35	53	184	12.963
Stomach	Men	86783	1185	9711	97680	216060	16587	220536	453183	550863	23.594
	Women	3017	41	398	3455	271	234	2757	3262	6718	8.221
Colorectum	Men	5563	76	576	6216	5834	1386	8704	15924	22140	22.525
	Women	<10	<10	<10	<10	389	<10	<10	389	389	NA
Liver	Men	88227	866	7660	96753	348640	1179	104903	454723	551476	56.356
	Women	6227	64	686	6976	4968	20	1903	6891	13867	18.781
Pancreas	Men	18471	223	2265	20960	79454	5099	12843	97396	118355	74.015
	Women	427	<10	63	494	218	35	89	342	837	20.923
Larynx	Men	18842	262	2180	21284	14800	4097	25315	44212	65497	18.723
	Women	447	<10	72	526	35	30	166	231	757	10.619
Lung	Men	196276	2349	21547	220172	579578	36586	129603	745767	965939	46.615
	Women	8904	106	1071	10080	9604	737	2721	13062	23142	22.337
Cervix uteri	Men	0	0	0	0	0	0	0	0	0	NA
	Women	435	7	47	489	2322	71	491	2884	3372	36.437
Ovary	Men	0	0	0	0	0	0	0	0	0	NA
	Women	3610	54	461	4126	2552	598	2091	5242	9368	22.942
Kidney	Men	3062	36	283	3381	4907	877	11029	16813	20195	22.498
	Women	<10	<10	<10	<10	198	<10	<10	198	198	NA
Bladder	Men	25327	448	3343	29117	21685	4968	49379	76032	105149	13.572
	Women	382	<10	62	451	133	23	203	359	810	7.586
Total		531642	6509	57165	595316	1438760	89119	637490	2165370	2760686	34.815

NA: not available because of very few patients.

The direct costs of cancers attributable to smoking were found to be nearly 595 million USD. In particular, lung, liver and stomach cancers were associated with the largest direct-cost expenditure, irrespective of gender. Noticeably, in men, lung, stomach and liver cancers were responsible for most of the direct healthcare costs, with nearly 70% of direct costs associated with these cancer types, whereas in women, around two-thirds of the total direct costs were associated with lung, liver and ovarian cancers. Nearly 90% of the estimated direct costs were from direct medical costs, with the remaining 10% from non-medical costs (e.g. travel costs to medical institutions and caregivers’ costs) (Table 2).

The indirect costs of smoking-attributed cancers were determined to be nearly three times higher than the direct costs, reaching approximately 2.2 billion USD. As with direct costs, the top three most costly smoking-related cancers in terms of indirect costs were lung, liver, and stomach cancers, both overall and for men. In women, lung cancer took the lead for the highest indirect costs, followed by ovarian and liver cancers (Table 2).

**Figure 1:**
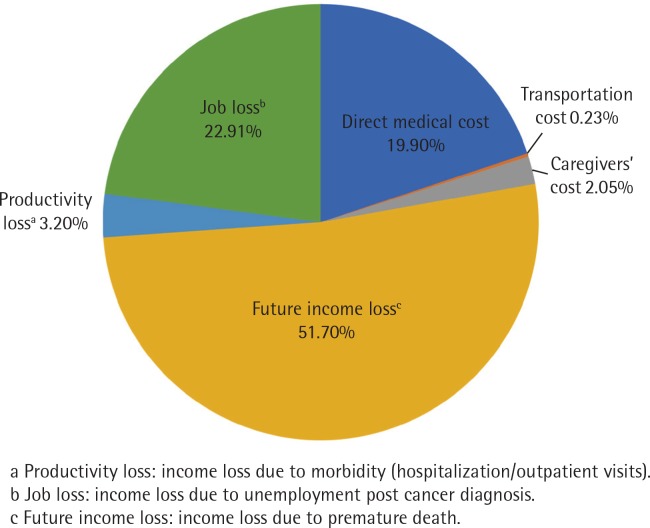
Proportions of cost components (%)

Nearly half of the total economic impact of smoking-related cancers stemmed from future income loss, followed by indirect costs due to job loss after cancer diagnosis and direct medical costs, at around 23% and 20%, respectively (Figure 1).

Sensitivity analysis was conducted applying discount rates of 0%, 3% and 5%. The economic burden increased by 12% with the no discount applied, reaching 3.12 billion USD, whereas the economic burden decreased to 2.59 billion USD when the 5% discount rate was applied (Table 3).

**Table 3:** Sensitivity analysis of economic burden of smoking-related cancers, applying discount rates from 0% to 5% (Thousand USD)

	*Discount rates*		
	*0%*	*3%*	*5%*
Indirect costs	2527206	2165370	1997761
Total costs	3122522	2760686	2593077

## DISCUSSION

The economic burden of cancers attributed to smoking in Korea in 2014 was approximately 2.76 billion USD, of which indirect costs were found to be significantly higher than direct costs by as much as 3.64 times. Previous studies also showed that indirect costs were more burdensome, reporting indirect costs as 2 to 3 times direct costs^20,32^. Furthermore, the economic burden of cancers attributable to smoking were 0.2% of the national GDP and about 1.19% of the total national healthcare expenditure in 2014^22,33^. It is worth noting that smoking-related cancers represented a less significant economic burden in 2014 compared to 2008 with 3104 million USD spent after adjusting for inflation, possibly resulting from a decreasing trend of cancer incidence and mortality^14^.

When the economic burdens of men and women are considered together, lung, liver and stomach cancers were the leading cause of economic costs. Among the major cancers in men, the costs of lung cancer were highest, followed by liver and stomach cancers, whereas in women the highest costs were associated with lung, liver, and ovarian cancers. These results are understandable in that lung, liver and stomach cancers have taken the lead in Korean mortality rates^34^. In the Oh et al.^20^ study, which estimated the economic burden of smoking-associated cancers in 2008, the top four cancers were (in decreasing importance) lung, liver, colorectal, and stomach cancers. The most noticeable difference is that in the current study stomach cancer replaced colorectal cancer as the third leading cause of cancer mortality, and colorectal cancer only accounted for a small proportion of total costs (0.82%) in our study population. One possible explanation for this is the difference in study methods and epidemiological characteristics used by each study. In our study, we employed the PAF of smoking in the Korean population from the Park et al.^21^ study, which reported that around 0.8% of all colorectal cancer cases are attributed to smoking regardless of sex, while the Oh et al. study calculated the PAF according to Levin’s formula, using *relative risk* from Japanese research^20^. This insignificant PAF value (i.e. 0.8%) is the primary reason for the small contribution of colorectal cancer to total costs incurred in our study.

It is interesting to note that the cost burden for men was nearly 39.3 times higher than for women in each cancer category, except of course for cervical and ovarian cancers. This is consistent with previous studies that reported total costs for men as being much higher than for women^20^. One reason for this is a huge difference in the PAFs for incidence and mortality that were used to calculate costs. According to the Park et al.^21^ study, there is a big gap in the PAF for smoking-related cancer incidence in men (20.9) compared with women (2.1) and a similar pattern in PAF for mortality. Also, our study results suggest that the number of smoking-attributed cancer cases was nearly 19 times higher in men (75325) compared to women (3972).

According to a study that systematically reviewed costs of smoking-related diseases, smoking-attributed illnesses were responsible for 1.5–6.8% of the national health expenditures and 0.22–0.88% of GDP for a given country^35^. Various studies investigating the economic burden of cigarette smoking-related cancers have been carried out in the United States and other high-income countries^36,37^. Given that smoking prevalence and cigarette consumption has been increasing dramatically in developing countries, where the burden of tobacco-related cancers is projected to increase by about 69.6%^38^, several studies have been conducted to highlight the rapidly rising burden of smoking-related diseases in those countries. For example, the economic costs of smoking in 2011 accounted for 0.97% of GDP in Vietnam, representing 5.76% of the government healthcare budget^39^, whereas in China, 3.1% of national healthcare expenditure was associated with smoking in 2000^40^.

In this study, job loss was included as an indispensable element of indirect costs. Although most studies that calculated the economic burden of smoking-related diseases did not take into consideration lost income associated with unemployment after cancer diagnosis, in Korea, job loss is a critical issue because of its large contribution to total costs. According to our study findings, job loss after a cancer diagnosis accounted for more than one-fifth of the overall smoking-associated cancer costs. It is very important to note the impact of cancer on patients’ working lives, since 5-year survival rates and mortality rates among Korean cancer patients have shown gradual signs of improvement^41^. One study reported that of 5396 patients engaged at baseline, a significant proportion (47%), were unemployed during a 3-year follow-up period^30^, suggesting that job loss drives a substantial portion of indirect costs.

Unlike other studies, our participants were identified by the V193 diagnostic code, which, in South Korea confers Expanded Benefit Coverage for patients with severe illnesses such as cancer and myocardial infarction. These expanded benefits helped to diminish extensive healthcare expenditures that might have occurred without the expanded coverage^42,43^. In contrast, several studies estimating the economic burden of cancers in Korea identified cancer patients by hospital admissions and number of outpatient visits. To be included in these studies, patients had to experience at least one inpatient admission and three outpatient visits^44^. One exception was a study on the economic costs of metabolic syndrome-related cancers^45^, for which special diagnostic codes were employed. Results of that study suggested that there was a slight difference in total costs when patients were identified by a frequency-of-visits definition versus the special diagnostic code definition. On that basis we can predict that there might be a modest effect in the total costs calculated in our study.

### Limitations

One limitation of our study concerns the possible overestimation of productivity loss resulting from premature death, which could result from using the human capital approach. This approach has been criticized for its assumption that a worker cannot be replaced even if unemployment is very high^46^. Nonetheless, the human capital approach is still one of the most frequently used methods to determine indirect costs of diseases. A second limitation stems from a potential underestimate of outpatient pharmaceutical costs, which cannot be verified in the NHIS claims data, and the costs for alternative and complementary medicine, which were not included in the estimates. Another possible shortcoming derives from the lack of several item cost estimations, of which might be program costs for prevention and early detection of smoking-related cancers, presentism and costs for cigarette purchases. Finally, the total cost of smoking-related cancers might also have been underestimated for several potential reasons: 1) only primary cancer diagnoses were included in this study; 2) cancers of the nasal cavity and accessory sinuses, ureter, and myeloid leukemia, which are known as a smoking-related cancers, were not included in the analysis because these cancers have low incidence and never reported Korean specific SAF; and 3) a positive association between smoking and breast cancer has been suggested but not included in this study because of lack of SAF.

Despite these limitations, this study provides valuable updated information on the economic burden of cancers associated with smoking in Korea.

## CONCLUSIONS

Despite the early initiation of tobacco control policy in Korea, the economic burden of cancers attributable to smoking remains high because cancer can take years to develop after the initial smoking exposure. In addition, the prevalence of smoking among young adults and women is increasing in Korea. Therefore, it is crucial that intense efforts are made to develop strict smoking control programs.

## CONFLICTS OF INTEREST

Authors have completed and submitted the ICMJE Form for Disclosure of Potential Conflicts of Interest and none was reported.
