# Supplementary material for: The economic burden of cancers attributable to smoking in Korea, 2014
Source: Tob Induc Dis. 2019 Feb 28;17:15. doi: 10.18332/tid/102673 (PMC6751966; doi:10.18332/tid/102673)
Supplement: Supplementary file 1 [file TID-17-15-s1.pdf]

Supplementary Table 1. Types of costs, description, and sources of data

**Type of costs**		**Description**	**Data sources**
Direct costs	Direct medical costs	Medical care covered by NHIS	NHIS claims data (2014)
		Medical care not covered by NHIS	Survey on the Benefit Coverage Rate of NHIS
	Direct non-medical costs		
	Transportation costs	One-way cost per visit	Korea Health Panel Survey (2014)
		Frequency of inpatient/outpatient visits	NHIS claims data (2014)
	Caregivers' costs	Days of inpatient admission	NHIS claims data (2014)
		Frequency of over-65-aged outpatients	NHIS claims data (2014)
		Caregivers' daily wage and utilization rate	Korea Health Panel Survey (2014)
Indirect costs	Future income loss	Number of cancer specific deaths	Cause of Death Statistics, Korea (2014)
		Employment rates	Ministry of Employment and Labor, Korea (2014)
		Life expectancy	Life tables, Statistics Korea (2014)
	Productivity loss	Frequency of inpatient/outpatient visits	NHIS claims data (2014)
		Employment rates	Ministry of Employment and Labor, Korea (2014)
		Average daily wage	Ministry of Employment and Labor, Korea (2014)
	Job loss	Number of cancer cases	NHIS claims data (2014)
		Job loss average rate	Park J.H. et al (2008)
		Employment rates	Ministry of Employment and Labor, Korea (2014)
		Average daily wage	Ministry of Employment and Labor, Korea (2014)

© 2019 Nguyen TXT
